# Supplementary material for: A cost description of the setup costs of community-owned maternity waiting homes in rural Zambia
Source: PLOS Glob Public Health. 2023 Apr 6;3(4):e0000340. doi: 10.1371/journal.pgph.0000340 (PMC10079123; doi:10.1371/journal.pgph.0000340)
Supplement: S1 Table — (DOCX) [file pgph.0000340.s002.docx]

**Supplementary Materials**

**S1 Table.** Overview of project-facilitated activities

| Activity | Goal of activity | Targeted Participants | Length of activity | Number of activities held | Frequency of activity | Average number of participants per activity^7^ |
| --- | --- | --- | --- | --- | --- | --- |
| Formation & capacity building of governance & management structures | | | | | | |
| Stakeholder Meeting | - Acquaint stakeholders with the project - Develop mission statement - Select Core Group representatives | - District-level stakeholders^3^ - Site-level stakeholders^4^ - Architects, contractor | 1 day | 1 meeting per district (4) | Once at start of project | 17 |
| Core Group Workshop | - Develop terms of reference for GC policies | - District-level stakeholders^3^ - Site-level stakeholders^4^ | 2 days | 1 meeting per district (4) | Once at start of project | 11 |
| GC Formation Meeting | - Train on principles of governance, mission statement, and terms of reference | - GC members (all) | 2 days | 1 meeting per district (3)^9^ | Once at start of project | 31 |
| GC Lessons Learned | - Discuss successes and challenges; facilitate cross- site learning | - GC members (selected)^6^ | 4 days | 1 meeting | Once mid-way through project | 40 |
| GC Mentorship^10^ | - Assess and troubleshoot challenges | - GC members | ~2 hours per visit | 1 visit per MWH site (10) | Monthly | Varied based on availability |
| MWH Annual General Meeting | - Foster accountability of the GC to the community - Increase ownership over MWH - Deliver MWH operational and financial reports - Election of committee members | - All GC members (host) - Site-level stakeholders^4^ - Community-level stakeholders^5^ | 1 day | 1 meeting per site (10)^8^ | Annually | 45  (includes GC members) |
| MU Induction Meeting | - Train on mission of the MWH and daily scope of work | - MU members (all) | 2 days | 1 training per site (10) | Once before opening of MWHs | 5  (1-10 per site) |
| MU Mentorship^10^ | - Discuss MWH operations and review register data | - MU members | ~2 hours per visit | 1 visit per MWH site (10) | Monthly | Varied based on availability |
| Stakeholder engagement | | | | | | |
| National Launch | - Engage national-level stakeholders to increase project buy-in | - National level stakeholders^1^ - Donors - Media | 1 day | 1 meeting | Once at start of project | 50  (estimated) |
| Headmen Engagement | - Generate commitment from traditional leadership to support general MWH maintenance and operations | - All GC members - Site level stakeholders^3^ - All headmen from within the catchment area | 1 day | 1 meeting per site (10) | Once at start of project | 35  (estimated) |
| Site launches | - Celebrate official openings of MWHs - Engage and educate local communities to increase buy-in - Entertainment and food provided. - Key stakeholders invited to speak to about the importance of maternal child health and facility-based deliveries | - Provincial level stakeholders^2,11^ - District level stakeholders^3^ - Site level stakeholders - Community level stakeholders | 1 day | 1 launch per site (10) | Once upon opening of MWH | 300 |
| Headmen Meeting | - Update traditional leadership on MWH maintenance and operations | - All GC members - Site level stakeholders^3^ - All headmen from within the catchment area | 1 day | 1 meeting per site (10) | Once mid-way through project | 35  (estimated) |
| Abbreviations: GC = Governance Committee; MU = Management Unit  *The activities above were held for the purpose of setup of the MWHs, establishment of the governance and management systems, and to garner early stakeholder support. While some activities may recur after the inception phase of the intervention, we do not analyze recurrent activities nor their running costs.  ^1^ National level stakeholders include: Ministries of Health and Community Development; NGOs in maternal and child health working nationally  ^2^ Provincial level stakeholders include: provincial permanent secretaries of health; provincial health officer  ^3^ District level stakeholders include: District health officers; community development officer; local chiefs or their representatives; relevant non-governmental organizations. For the site launches only this also includes: Agriculture development officers, traditional affairs officers, and district councilors  ^4^ Site-level stakeholders include: health center staff & community-based volunteers  ^5^ Community-level stakeholders include: local religious, civic, and traditional leaders (headmen); other interested community members  ^6^ Selected GC members often included: GC Chairperson, Treasurer, Secretary, and specific subcommittee members  ^7^ Attendance figures for some activities were not available. In these instances, best estimates were included.  ^8^ Some or all sites may require preparatory meetings with project staff to ensure readiness for the Annual General Meeting.  ^9^ Though this project includes 4 districts, only 3 meetings were held for the GC formation. Participants from the two smaller, contiguous districts of Choma and Pemba participated in 1 meeting.  ^10^ GC and MU mentorship were generally conducted during the same visit to maximize efficiencies for transport and human resources time  ^11^ One MWH site launch was much larger than the rest and included more provincial-level stakeholders and media personnel | | | | | | |
