# Supplementary material for: A cost description of the setup costs of community-owned maternity waiting homes in rural Zambia
Source: PLOS Glob Public Health. 2023 Apr 6;3(4):e0000340. doi: 10.1371/journal.pgph.0000340 (PMC10079123; doi:10.1371/journal.pgph.0000340)
Supplement: S2 Table — (DOCX) [file pgph.0000340.s003.docx]

**S2 Table.** Main structural elements and furnishing for the MWHs provided by the MAHMAZ project

|  | **Structural Elements** | **Furnishings** |
| --- | --- | --- |
| Infrastructure | Concrete walls and floors  Sheet metal roof  Glass windows with locks  Wooden doors with lockable metal handles  5 exits with concrete ramps | 5 solar lights  9 bulbs  22 window curtains  11 curtain rods |
| Sleeping | 1 large dormitory  1 small dormitory | 14 wooden, non-hospital grade beds  28 plastic-covered mattresses  37 plastic-covered pillows  14 mosquito nets  14 metal cabinets for personal belongings with locks  26 sheet sets  14 comforters  28 blankets  26 pillowcases |
| Hygiene | Two sinks | 2 large plastic water drums  3 hand washing buckets with spigots and stands  2 toilet brushes  Basic cleaning products (for washing clothes, washing floors, handwashing) |
| Sanitation | 3 latrines (1 site had flushable toilets)  3 stalls for bathing | 3 shower curtains with rods |
| Laundry | 1 large walled courtyard with drying racks | 1 large plastic washing tub  4 poles with wire (to hang clothes outside) |
| Cooking | Small, covered cooking shelter | 3 metal pots and pans  Metal cooking utensils (2 knives, 10 spoons,)  6 wooden cooking spoons  2 mixing bowls  14 plastic plates  13 plastic cups  2 metal braziers  2 high efficiency wood-burning stoves  2 food warming containers |
| Administration | Small office  Wooden shelving unit | 1 wooden desk  1 wooden table  2 wooden chairs  2 metal/wooden file cabinets  2 mops  2 brooms  Framed certificate of occupancy  Poster board with mission and vision statements  Poster board with patient rights and responsibilities  1 metal safe  1 electronic calculator  1 MWH utilization register |
| Recreation | Open-air verandah  Concrete benches along 3 walls | 1 large white board  1 box of white board markers (11 markers) |
