# Supplementary material for: A cost description of the setup costs of community-owned maternity waiting homes in rural Zambia
Source: PLOS Glob Public Health. 2023 Apr 6;3(4):e0000340. doi: 10.1371/journal.pgph.0000340 (PMC10079123; doi:10.1371/journal.pgph.0000340)
Supplement: S3 Table — (DOCX) [file pgph.0000340.s004.docx]

**Supplementary Table 3.** Univariate sensitivity analysis of annuitized financial costs incurred by MAHMAZ project, using variable discount rates

| Cost Categories | Actual Cost | Annuitized Costs | | | | Range* |
| --- | --- | --- | --- | --- | --- | --- |
|  |  | 2% Discount Rate | 3% Discount Rate | 5% Discount Rate | 10% Discount Rate |  |
| Capital Costs | | | | | | |
| Infrastructure | $53,240 | $2,377 | $2,716 | $3,463 | $5,648 | $3,271 |
| Furnishing | $11,311 | $2,400 | $2,470 | $2,613 | $2,984 | $584 |
| Subtotal | **$64,551** | **$4,777** | **$5,186** | **$6,076** | **$8,631** | **$3,855** |
| Installation Costs | | | | | | |
| Capacity Building | $12,336 | $4,278 | $4,361 | $4,530 | $4,960 | $683 |
| Stakeholder Engagement | $8,397 | $2,912 | $2,969 | $3,084 | $3,376 | $465 |
| Subtotal | **$20,733** | **$7,189** | **$7,330** | **$7,613** | **$8,337** | **$1,148** |
| Grand Total | **$85,284** | **$11,966** | **$12,516** | **$13,689** | **$16,968** | **$5,002** |
| *Range calculated by subtracting annuitized price using 2% discount rate from the annuitized price using 10% discount rate | | | | | | |
