# Supplementary material for: A cost description of the setup costs of community-owned maternity waiting homes in rural Zambia
Source: PLOS Glob Public Health. 2023 Apr 6;3(4):e0000340. doi: 10.1371/journal.pgph.0000340 (PMC10079123; doi:10.1371/journal.pgph.0000340)
Supplement: S4 Table — (DOCX) [file pgph.0000340.s005.docx]

**Supplementary Table 4.** Theoretical costs assuming variable inflation rate of infrastructure costs

| Cost Categories | Actual Costs | Theoretical Costs | |
| --- | --- | --- | --- |
|  |  | 25% inflation of infrastructure costs | 50% inflation of infrastructure costs |
| Infrastructure | $53,240 | $66,550 | $79,860 |
| Furnishing | $11,311 | $11,311 | $11,311 |
| Capital Costs | **$64,551** | **$77,861** | **$91,171** |
| Capacity Building | $12,336 | $12,336 | $12,336 |
| Stakeholder Engagement | $8,397 | $8,397 | $8,397 |
| Installation Costs | **$20,733** | **$20,733** | **$20,733** |
| Grand Total | **$85,284** | **$98,594** | **$111,905** |
