# Supplementary figures and images for: A cost description of the setup costs of community-owned maternity waiting homes in rural Zambia
Source: PLOS Glob Public Health. 2023 Apr 6;3(4):e0000340. doi: 10.1371/journal.pgph.0000340 (PMC10079123; doi:10.1371/journal.pgph.0000340)

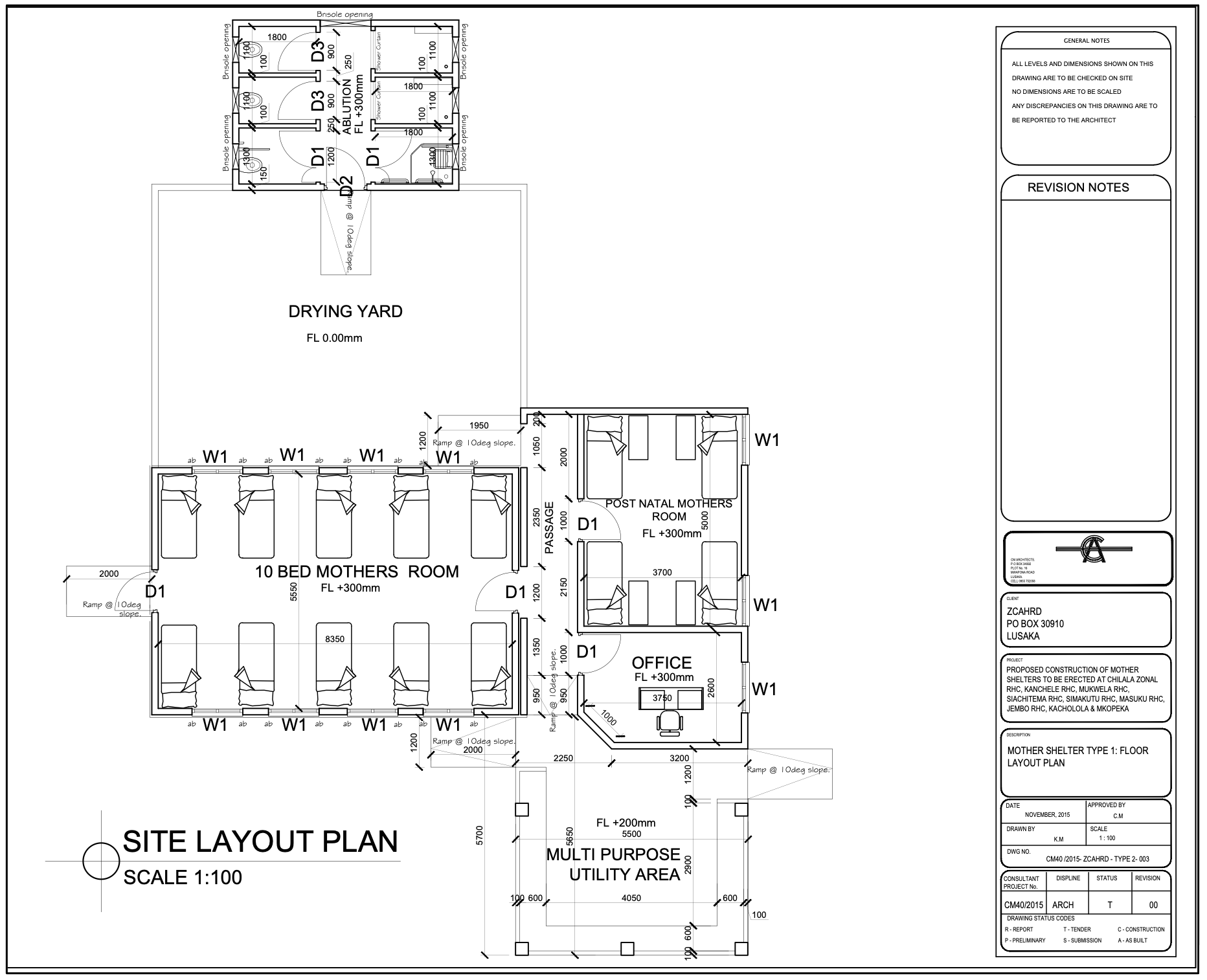

Supplement: S1 Fig — (TIF) [file pgph.0000340.s006.tif]

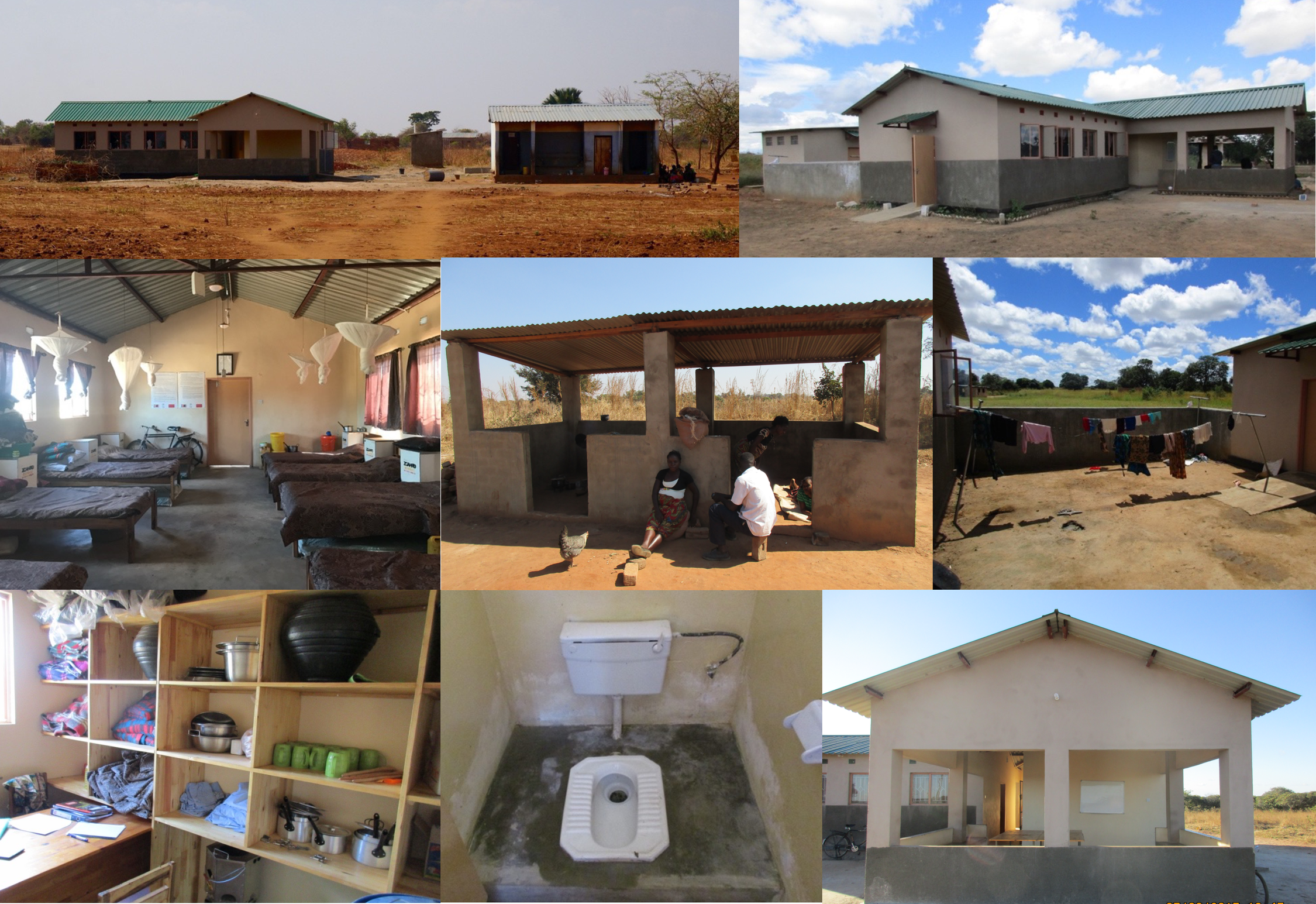

Supplement: S2 Fig — Components from left to right, top to bottom: a. New and old MWH structures; Credit: Jeanette Kaiser, Boston University School of Public Health, 2017. b. New MWH structure; Credit: Jeanette Kaiser, Boston University School of Public Health, 2017. c. Main dormitory with 10 beds, bedding, mosquito nets, and cabinets; Credit: Wakunyambo Imasiku, Zambia Center for Applied Health Research and Development, 2017. d. Cooking shelter; Credit: Oliver Malupande, Zambia Center for Applied Health Research and Development, 2017. e. Courtyard and drying racks; Credit: Jeanette Kaiser, Boston University School of Public Health, 2017. f. Office and storage room with a desk, cooking pots, cups, and bedding; Credit: Jeanette Kaiser, Boston University School of Public Health, 2017. g. Latrines; Credit: Jeanette Kaiser, Boston University School of Public Health, 2017. h. Verandah; Credit: Wakunyambo Imasiku, Zambia Center for Applied Health Research and Development, 2017. (TIF) [file pgph.0000340.s007.tif]
