# Supplementary material for: A cost description of the setup costs of community-owned maternity waiting homes in rural Zambia
Source: PLOS Glob Public Health. 2023 Apr 6;3(4):e0000340. doi: 10.1371/journal.pgph.0000340 (PMC10079123; doi:10.1371/journal.pgph.0000340)
Supplement: Attachment — Submitted filename: PGPH-D-22-00186_Response to reviewers 18Oct2022.docx [file pgph.0000340.s008.docx]

**Response to Reviewer Comments**

**A cost description of the setup costs of community-owned maternity waiting homes in rural Zambia**

*Manuscript PGPH-D-22-00186*

18 October 2022

1. We note your Data Availability Statement: "Data are available in a public, open access repository. De-identified data for this analysis will be made available through OpenBU. The OpenBU repository has data access policies and procedures consistent with NIH data sharing policies."

Unfortunately, this information (<https://open.bu.edu/>) does not lead to your dataset. Please provide the correct DOI/link and that your data is set to be publicly available. We hope to hear from you soon with updated information.

**Thank you for this comment. The following link leads to our datasets:** <https://hdl.handle.net/2144/45243>**. They will be embargoed until the manuscript is publicly available, but we can grant access as necessary.**
