# Supplementary material for: A cost description of the setup costs of community-owned maternity waiting homes in rural Zambia
Source: PLOS Glob Public Health. 2023 Apr 6;3(4):e0000340. doi: 10.1371/journal.pgph.0000340 (PMC10079123; doi:10.1371/journal.pgph.0000340)
Supplement: Attachment — Submitted filename: PGPH-D-22-00186R2_Response to reviewers.docx [file pgph.0000340.s010.docx]

Reviewer's Responses to Questions

**Comments to the Author**

| **Reviewer 2** | |
| --- | --- |
| 1. The first has to do with the sensitivity and scenario analysis. Ideally the authors would put their scenario and sensitivity analysis in one sub-section, describing them each to help understand the effect of these on the results, and the robustness of their results. In the methods section, they would move the description of changes in discount rate (line 279-281) and changes in infrastructure costs (lines 266-268) to the paragraph where they also vary assumptions on occupancy (lines 298 to 305). The sub-header could read Sensitivity and Scenario analysis.  a. The authors conduct a univariate sensitivity analysis of the discount rate, varying it to 2% and 5%. 3% is the low end, and I would recommend redoing that at a more realistic rate of 5% and 10%.  b. Then in results section, they would present their base case first, followed by estimates from the scenario and sensitivity analysis. I’d recommend moving lines 340 to 345 to around lines 365, when they also present the variation in occupancy. | Thank you for this comment. We restructured the methods section to put the description of these theoretical scenarios under one heading (page 13).  a. When we conducted the analysis, we felt most comfortable using a 3% discount rate. We continue to feel inclined to provide estimates that are lower and higher than this rate, and have taken the reviewer’s advice, adding a column to Supplementary Table 3 to show the 10% discount rate.  b. We have taken the reviewer’s suggestion. We’ve moved the paragraph discussing our sensitivity and scenario analyses to the end of the results section, following the description of the theoretical occupancy (page 17). |
| 2. Table 2 headers. Need to indicate that results presented in USD. Rather than add (%of total cost) in the column header, may want to put a footnote indicate that the numbers in parenthesis are % of total cost), otherwise the table is a little confusing as edited. | Thank you for this comment. We edited the table title and added an additional footnote to describe the values in parentheses. |
| **Reviewer 3:** | |
| ABSTRACT: #MAHMAZ in line 26 should be written in full form at the first appearance of the short form. | Thank you, we have made this change. |
| INRODUCTION: #Author has used "." sign before the reference (e.g., ".(1-3)" in line 52). This must be revised. The full stop sign (.) must be used after the reference (i.e., "(1-3)."). This issue should be checked for the full manuscript. | Thank you, we corrected this formatting throughout the manuscript. |
| METHODS: #Line 84- "MAHMAZ situated the new...…." here, situated should be replaced by "established". | We have revised this wording. |
| METHODS: #Line 119- unnecessary gap should be removed. | Thank you, I believe we have removed this gap. |
| METHODS: #Line 222- "These costs are not are not included..." sentence should be corrected by removing "are not" for once. | Thank you for catching this typo. We removed one of the duplicate “are not”s. |
| RESULTS: #The description and the value of the tables are not matching. The inconsistency was found in full result section. Either the tables or the descriptions have problems. This inconsistency must be met properly. | Thank you.  We corrected the range. We had inadvertently used the range between discount rates for the Capital costs subtotal ($1,299). The document now reflects the correct range, $1,709.  We also adjusted the values in Figure 1. The values stayed the same, but we made the lower left hand figure round numbers instead of decimals. |
| DISCUSSION: # The whole discussion section should be checked for the standard of the English language that has been used in the manuscript. | Thank you. We have reviewed the discussion section for grammar and language. |
