# Supplementary material for: A cost description of the setup costs of community-owned maternity waiting homes in rural Zambia
Source: PLOS Glob Public Health. 2023 Apr 6;3(4):e0000340. doi: 10.1371/journal.pgph.0000340 (PMC10079123; doi:10.1371/journal.pgph.0000340)
Supplement: Attachment — Submitted filename: PGPH-D-22-00186_Response to Reviewer Comments 121222.docx [file pgph.0000340.s009.docx]

**Response to Reviewer Comments – 12 December 2022**

**A cost description of the setup costs of community-owned maternity waiting homes in rural Zambia**

*Manuscript PGPH-D-22-00186*

1. Please amend your detailed Financial Disclosure statement. This is published with the article. It must therefore be completed in full sentences and contain the exact wording you wish to be published.

a. State the initials, alongside each funding source, of each author to receive each grant. For example: ""This work was supported by the National Institutes of Health (####### to AM; ###### to CJ) and the National Science Foundation (###### to AM).""
b. If any authors received a salary from any of your funders, please state which authors and which funders.

If you did not receive any funding for this study, please simply state: “The authors received no specific funding for this work.”

**Thank you for this comment. I have revised the Financial Disclosure statement, including the initials of the authors who received funding to each respective grant.**

2. We notice that your revision was submitted on Dec 8, 2022, but the manuscript file in your submission's file inventory was uploaded on Sep 16, 2022. Please upload the latest version of your revised manuscript as the main article file, with the item type 'Manuscript,' ensuring that it does not contain any tracked changes or highlighting. This will be used in the production process if your manuscript is accepted.

**Thank you. We have uploaded the latest version of our revised manuscript.**

**Response to Reviewer Comments – 8 December 2022**

**A cost description of the setup costs of community-owned maternity waiting homes in rural Zambia**

*Manuscript PGPH-D-22-00186*

1. Thanks for the revised version. I did not find any responses regarding the reviewers raised in original version of the manuscript. Please respond accordingly.

**Thank you for this comment. I have attached the full track-changes version of the manuscript, along with all responses to reviewers, below.**

**Response to Reviewer Comments – 4 September 2022**

**A cost description of the setup costs of community-owned maternity waiting homes in rural Zambia**

*Manuscript PGPH-D-22-00186*

| **Reviewer 1** |  |
| --- | --- |
| In the title the authors indicated that they did an economic evaluation which was not revealed in the objective and rest of the paper. The authors may refer books and articles to understand and conduct an economic evaluation. There are full and partial economic evaluation. The book written by Michael F. Drummond and his colleagues is a good book for anyone interested to conduct an economic evaluation of healthcare programs. You may follow the link: <https://books.google.com.et/books/about/Methods_for_the_Economic_Evaluation_of_H.html?id=yzZSCwAAQBAJ&redir_esc=y> | Thank you for this. We agree that Drummond’s work is foundational to economic evaluation and was included as a reference (23). We’ve clarified that this is a cost analysis: on page 3 we detailed that this is a partial, top-down analysis of financial costs. We have also changed the title: *“A cost analysis of the setup costs of community-owned maternity waiting homes in rural Zambia”* |
| A good article by Luke Rudmik, and Michael Drummond, published in 2013 defines economic evaluation as: “…is the comparative analysis of alternative courses of action in terms of both their costs and consequences. With increasing health care expenditure and limited resources, it is important for physicians to consider the economic impact of their interventions.” https://onlinelibrary.wiley.com/doi/10.1002/lary.23943 However, there is nothing said about consequence in the study’s contents. Even the costs described in the study are simple reports of set-up costs. The core thing in research, which is a systematic search some issue to establish facts and reach new conclusions were not addressed sufficiently in the study. The authors simply collected some financial data and presented that in a table. | We have reworded our title and methods to state that this is a cost analysis.  This is a presentation of our implementation data. We present our financial costs and aim for this work to serve as a reference for implementers conducting similar work. |
| The authors may collect additional relevant data about service costs, users' costs and some measure of consequences to improve their study. It would be better if they take some comparator in their analysis, even do nothing could be a comparator. | We agree that service costs and users’ costs are useful, but not what we set out to do in this work. We cited other studies which analyze user costs, etc. and we find them useful and agree with them, however, we found that setup costs for this type of implementation has not been well reported and we aim to fill a gap in the literature. |
| **Reviewer 2** |  |
| 1. Page 12, line 242, I’d recommend that the authors use the term ‘Start-up’ costs relating to one-time, non-recurring activities, rather than ‘implementation costs’. Implementation of an intervention can occur during a start-up and on-going phases. Start-up costs are a common term used in global health costing studies. If they want to refer to one-time, non-recurring activities as something other than ‘Start-up’, I’d suggest, ‘Installation’, rather than ‘Implementation’. Typical ‘Start-up’ cost categories are infrastructure and furnishing, capacity building (training), stakeholder engagement. Others may include materials development, for example. They could omit the foot note in table 2, if they used the terminology ‘Start-up’ costs or ‘Installation’ costs. IF changed, they would need to change headers in Figure 1 and in other places in the text. | Thank you for this suggestion. We changed the verbiage from ‘implementation costs’ to ‘installation costs’ throughout the paper. We often use the phrase setup, and start up may be too confusing, but we agree that installation is an appropriate term here. |
| 2. Page 12, lines 252-253: These two sentences could use a little rewording. “We present costs as an average per site. In many instances costs were recorded at the program level and could therefore not be allocated to specific sites. Costs by category were therefore evenly allocated across sites by dividing total costs by the number of sites (10). “   a. “Costs as an average per site, or do you mean ‘average set up costs’—i.e. sum of total set up costs across all sites divided by number of sites. I think the authors derive an average across all sites. This is just an average cost for setting up the maternity waiting home. b. They indicate that many costs couldn’t be allocated to specific sites, which is expected. Were ALL costs allocated across sites, or just some of the shared program costs? If the latter could they add a little descriptive, such as “For example, some shared program costs, such as x, y and z, were allocated evenly across each site by dividing the total shared program costs by the number of sites (10 sites). These costs were then added to the site-specific costs. (Or something to that effect) | a. Thank you for noting this. We reworded this phrase to simplify the message and indicate that we allocated the costs over the 10 sites.  b. Thank you. We added a sentence to provide an example: “*For example, the primary labor contract was presented as one flat fee, not broken up by site; This cost was then allocated across the 10 sites to get a site-level cost.”.* |
| 3. Page 13, line 280-286—I’m not sure why the authors refer to cost per admission and cost per bed-night, since these costs do not include operating costs. Cost per bed-night would typically include operating costs. | Thank you for these comments. We included clear language describing that cost per visit and cost per bed-night are based solely on startup costs and exclude any operating costs. We also describe our rationale for including these costs, “*We display these costs to highlight how this component of future costs may change with different levels of utilization.”* We believe that implementors may find value in modeling occupancy scenarios and that it may help to inform their own work.  We have included a footnote to Table 3 to reiterate that operating costs are not included in this analysis. |
| 4. I think it is fine to present a unit cost based on different scenarios of utilization, but just confused by reference to cost per admission and cost per bed-night. Can the authors present a clearer rationale for this, or is it possible to simply refer to ‘Cost per MWH visit’ under different occupancy scenarios and calculations? |  |
| 5. The authors do not conduct a sensitivity analysis, and it would make the results more generalizable to other settings if they could do this. At a minimum, it would be useful to vary the discount rate of 3%, since for capital infrastructure investments, this assumption is critical. If there are any other uncertain variable estimates, they could vary those as well. I wonder if they could consider international vs local prices for key construction materials, or consider varying labor costs, if Zambia construction labor is much higher or lower than other Southern African countries. I don’t think it would be a heavy lift to do univariate sensitivity analysis for this paper.  a. The authors note in the discussion that the constructions costs were significantly lower than other published estimates for Zambia, so sensitivity analysis may help to strengthen robustness of the authors results. | Thank you for this comment. We agree that this will provide important context. We have included two supplementary tables to consider different theoretical situations.   ST3 presents the actual project costs and annuitized costs considering 2%, 3%, and 5% discount rates.  ST4 presents two additional situations considering infrastructure costs inflated by 25% and 50%. |
| 6. Minor: Indicate somewhere in the paper, you are analyzing Financial costs. (i.e. you do not include economic or opportunity costs in this analysis).  Also indicate what year USD are in, i.e. 2020 USD or? All tables and figures should clearly indicate the USD (year) in the table or figure title. | Thank you. I clarified in line 76, “Here we present the direct *financial* costs required…”  Thank you for this comment. We have converted all costs to 2022 USD. We revised the language in our methods section accordingly: *“All expenses were recorded in Zambian Kwacha (ZMW) at the time of procurement. During analysis, costs were inflated to reflect 2022 costs. Finally, they were adjusted to US Dollars (USD), using the average exchange rate from the first 6-months of 2022.*” |
| Results 1. Page 14, line 311. Should this read, “The average cost to set up one MWH….”  a. Do the authors have disaggregated costs by site, so they could include that in an appendix table? This would show the site-specific costs for each site, and then the allocated shared program costs. This is related somewhat to the above question on how average costs were estimated.  b. Lines 313 to 315 provide cost shares, however neither Table 2 or Figure 1 show this. This sentence is confusing:  “The infrastructure costs made up the majority (62%) of the actual project costs but comprised only 21% of actual costs when annuitized.” I think it may read better as: The infrastructure costs made up the majority (62%) of the actual project costs but comprised only 21% of costs when annuitized.” (DELETE ‘ACTUAL’)  c. I’d recommend that in Table 2, the authors add (cost shares, % of total cost) in parenthesis in each column where cost data are presented: Planned cost per site, Actual Cost per site and Annuitized cost. Then the reader could see this more clearly. | Thank you, we corrected this wording.  We are not able to present disaggregated costs as an appendix at this time, however, we have clarified that costs were allocated down to the site level.  Thank you, we corrected this wording as suggested  I added the share of total cost to this table. |
| Discussion 1. Page 20, lines 411-414, this is confusing. Did the authors present their costs to a study in Ethiopia? I may suggest deleting lines 411 to 414 or elaborating a bit more. | Thank you for sharing this. We did not present our costs to this Ethiopia study. We reworded this section to clarify our message. |
| 2. Kudos to the authors for anchoring the results within the context of the Zambian MOH budget. Nicely done! | Thank you! |
| 3. Lines 415 to 446 could likely be synthesized a bit more and shortened, i.e. there are number of systematic reviews aimed at comparing costs to strengthen maternal and child health systems in LMIC, but the studies are either not comparable nor have they published their results. In addition, few report start up programmatic costs (43, 44, 45, and ?)  a. Minor: Line 440, reference for systematic review? | Thank you for this comment. We shortened this paragraph, describing that while some research has been done, there is little that is comparable to the present study.  Thank you, I added this reference |
| Suggestion: The authors may want to check their analysis against the Global Health Cost Consortium Reference Case check list. I think they adhere to it, with one or two exceptions. Available on line https://ghcosting.org/pages/standards/reference_case | Thank you for sharing this tool. We reviewed it and agree, that we mostly adhere to its recommendations. We have referenced this tool throughout our paper. |

**Response to Reviewer Comments – 15 September 2022**

**A cost description of the setup costs of community-owned maternity waiting homes in rural Zambia**

*Manuscript PGPH-D-22-00186*

| **Reviewer 1** |  |
| --- | --- |
| 1. Thank you for providing the following Data Availability Statement: "Data are available in a public, open access repository. De-identified data for this analysis will be made available through OpenBU. The OpenBU repository has data access policies and procedures consistent with NIH data sharing policies."  Before we can proceed, please provide us with the contact information/URL for OpenBU. | The URL for OpenBU is as follows: <https://open.bu.edu/>. We have also included this link in our cover letter. |
| 2. Please include a complete copy of PLOS’ questionnaire on inclusivity in global research in your revised manuscript. | We have completed the questionnaire and included it as supporting information in our submission. |
| 3. Please provide separate figure files in .tif or .eps format only and ensure that all files are under our size limit of 10MB. | Thank you for sharing instructions for file conversion via the PACE tool. We have converted all three of our files to .tif format. |
| 4. We noticed that you used “unpublished data” in the manuscript. We do not allow these references, as the PLOS data access policy requires that all data be either published with the manuscript or made available in a publicly accessible database. Please amend the supplementary material to include the referenced data or remove the references. | Thank you for this comment. We removed the references to unpublished data and replaced them with published articles. |
| 5. We do not publish any copyright or trademark symbols that usually accompany proprietary names, eg (R), (C), or TM  (e.g. next to drug or reagent names). Please remove all instances of trademark/copyright symbols throughout the text, including ® (SurveyCTO®, Microsoft®) on pages 12 and 13. | We have removed copyright symbols from our text. |
| 6. We have amended your Competing Interest statement to comply with journal style.  “The authors have declared that no competing interests exist.” | Thank you for making this change. |

**Response to Reviewer Comments – 18 October 2022**

**A cost description of the setup costs of community-owned maternity waiting homes in rural Zambia**

*Manuscript PGPH-D-22-00186*

18 October 2022

1. We note your Data Availability Statement: "Data are available in a public, open access repository. De-identified data for this analysis will be made available through OpenBU. The OpenBU repository has data access policies and procedures consistent with NIH data sharing policies."

Unfortunately, this information (<https://open.bu.edu/>) does not lead to your dataset. Please provide the correct DOI/link and that your data is set to be publicly available. We hope to hear from you soon with updated information.

**Thank you for this comment. The following link leads to our datasets:** <https://hdl.handle.net/2144/45243>**. They will be embargoed until the manuscript is publicly available, but we can grant access as necessary.**
